# The development and application of a novel E-commerce recommendation system used in electric power B2B sector

**DOI:** 10.3389/fdata.2024.1374980

**Published:** 2024-07-31

**Authors:** Wenjun Meng, Lili Chen, Zhaomin Dong

**Affiliations:** ^1^School of Computer Science and Technology, Beijing Institute of Technology, Beijing, China; ^2^Beijing Huadian E-commerce Technology Co., Ltd, Beijing, China; ^3^School of Public Health, Southeast University, Nanjing, China; ^4^School of Materials Science and Engineering, Beihang University, Beijing, China

**Keywords:** B2B, electric power industry, recommendation system, data fusion, user behavior

## Abstract

The advent of the digital era has transformed E-commerce platforms into critical tools for industry, yet traditional recommendation systems often fall short in the specialized context of the electric power industry. These systems typically struggle with the industry's unique challenges, such as infrequent and high-stakes transactions, prolonged decision-making processes, and sparse data. This research has developed a novel recommendation engine tailored to these specific conditions, such as to handle the low frequency and long cycle nature of Business-to-Business (B2B) transactions. This approach includes algorithmic enhancements to better process and interpret the limited data available, and data pre-processing techniques designed to enrich the sparse datasets characteristic of this industry. This research also introduces a methodological innovation that integrates multi-dimensional data, combining user E-commerce activities, product specifics, and essential non-tendering information. The proposed engine employs advanced machine learning techniques to provide more accurate and relevant recommendations. The results demonstrate a marked improvement over traditional models, offering a more robust and effective tool for facilitating B2B transactions in the electric power industry. This research not only addresses the sector's unique challenges but also provides a blueprint for adapting recommendation systems to other industries with similar B2B characteristics.

## 1 Introduction

**Figure 1:**
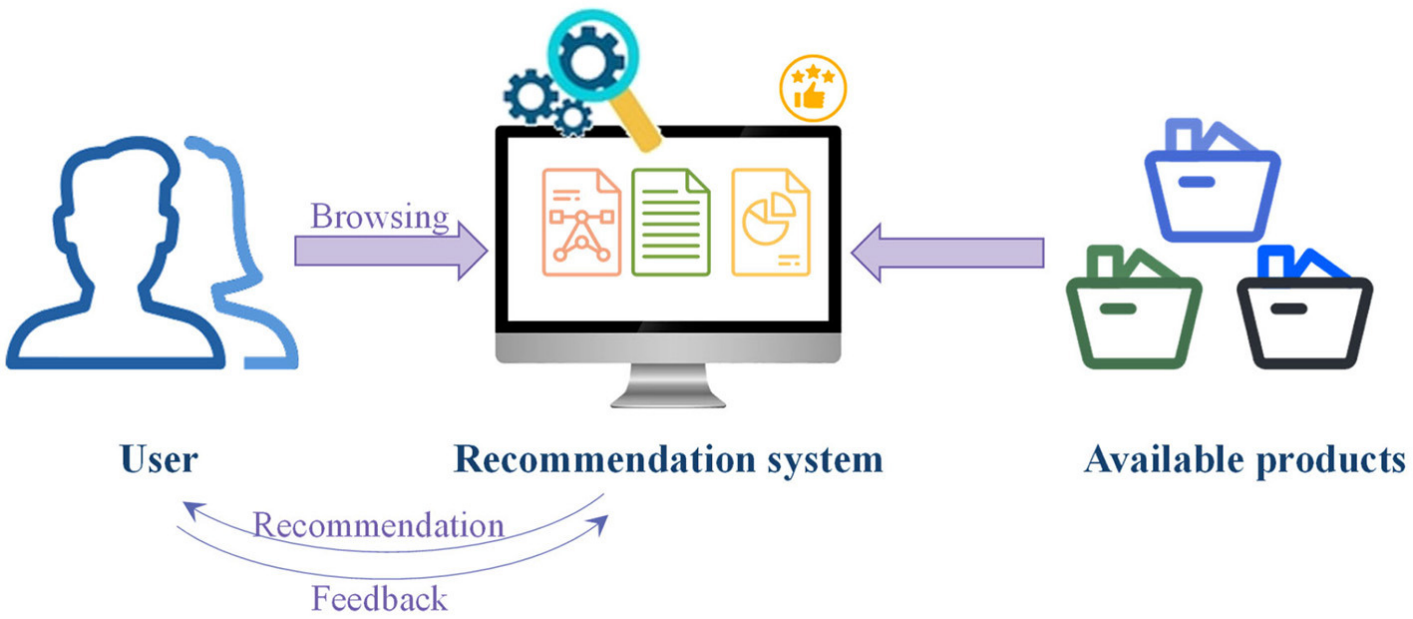
The diagrams for E-commerce ecosystem.

In the past two decades, the advent of E-commerce has transformed the commercial landscape, introducing platforms that are now integral to the consumer and business sectors alike. Developing a dependable and efficient recommendation system tailored to individual industries is crucial for advancing business activities. These advanced recommendation systems have proven invaluable in optimizing user experiences and boosting sales (Zhou et al., 2023), as depicted in Figure 1. While E-commerce principles are widely recognized, the specific challenges and nuances of each industry call for tailored solutions (Alduaij and Alterkait, 2022). The electric power industry, in particular, presents a unique case with its own complexities and demands, which are yet to be fully addressed by existing recommendation systems.

The electric power sector is characterized by its dependence on heavy machinery and equipment, which necessitates prolonged procurement cycles and intricate decision-making. These B2B transactions differ markedly from those in other industries due to the substantial capital investments required, stringent safety standards, and the need for precise technical specifications. The infrequent yet critical nature of these transactions creates a challenge for data collection and the development of accurate recommendation systems. The products in this industry are not only limited in number but also highly specialized, each with a detailed set of specifications, compliance requirements, and often, industry-specific certifications. A recommendation system for this sector must, therefore, be sophisticated enough to navigate these complexities and offer solutions that align with the technical and regulatory specifics of the industry. Hence, in stark contrast to traditional B2C (Business-to-Customer) models that dominate E-commerce, the electric power industry operates primarily within a B2B (Business-to-Business) framework. Here, transactions are not impulsive but are the result of meticulous planning and strategic decision-making, reflecting the significant financial stakes and operational implications involved. The recommendation systems prevalent in B2C platforms are ill-suited for the electric power sector, where the purchase cycles are longer, the stakes higher, and the products far more specialized. In addressing the inquiries related to the differentiation of users in a B2C context, it is crucial to underscore that, within our study's framework, the “users” are companies engaging in the procurement of specialized equipment, not individual end-consumers. The “items” are the products purchased by “users,” which is highly relevant to their business of companies. This distinction is pivotal as each company operates through a single account, eliminating scenarios of multiple individuals sharing one account or multiple accounts associated with a single entity. This unique characteristic of our B2B environment underscores the tailored approach required in developing recommendation systems that cater to the nuanced needs of these corporate entities, distinguishing our work from typical B2C applications where individual consumer preferences predominate.

Recognizing the absence of specialized recommendation systems in the electric power industry, this paper proposes an innovative approach. This manuscript aims to develop an integrated recommendation engine tailored for the E-commerce platforms specific to this sector. By synergizing multi-dimensional data streams (such as detailed user behavior patterns, product specifications, and broader industry trends) and leveraging advanced machine learning techniques, we intend to address the unique challenges of B2B transactions in the electric power industry. In particular, to comprehensively tackle these challenges, we propose an integrated recommendation engine specifically designed for the E-commerce platforms of the electric power industry. The main contributions of this study can be summarized as follows:

1) Industry specificity: this is the first attempt to develop a recommendation system tailored for the B2B context of the electric power sector. The challenges posed by low frequency, long duration transactions, and the scarcity of consistent user behavior data in E-commerce platforms are addressed head on.
2) Multidimensional data fusion: the approach integrates a variety of data sources, including user E-commerce activity, product descriptions, and essential user information like non-tendering data. This comprehensive data fusion ensures a holistic understanding of user preferences and needs.
3) Recommendation model integration engine: the core of this system is an advanced engine that combines offline recall models, multi-dimensional similarity calculations, and ensemble learning mechanisms. This integration ensures that the recommendations are not only accurate but also adaptable to the dynamic nature of the industry.

The rest of this article is organized as follows. Section 2 provides the literature review on E-commerce recommendation. The proposed methodology is stated in Section 3. The proposed approach is validated through a real case in Section 4. Finally, the conclusions of the article are presented in Section 5.

## 2 Literature review

### 2.1 E-commerce recommendation systems

In the rapidly evolving world of E-commerce, recommendation systems have emerged as a cornerstone, profoundly influencing online shopping experiences. These systems, adept at aligning products and services with nuanced user preferences, have been the focal point of numerous research endeavors, each contributing unique insights while also highlighting challenges that remain to be addressed.

Schafer et al. (2001) stands as a testament to the early innovations in the field. Providing a comprehensive taxonomy of recommender systems, the study juxtaposes them with traditional database analysis techniques, offering a holistic perspective. However, given the rapid technological advancements since its publication, some of its methodologies and insights might now be overshadowed by newer, more advanced innovations. Wang et al. (2018) is noteworthy for its solutions addressing scalability, sparsity, and the cold start conundrums in recommendation systems. Yet, the platform specific nature might limit its wider applicability. Chen et al. (2019) stands out for its innovative use of the transformer model to capture sequential user behavior signals, showcasing a superior performance benchmark. However, the model's specificity to Alibaba's platform raises questions about its generalizability.

Ebrahimi et al. (2019) delves deep into the symbiotic relationship between trust and customer satisfaction. Their research underscores the pivotal role of recommendation systems in fortifying and enhancing customer loyalty, a critical metric in today's competitive E-commerce environment. However, a potential shortcoming of their work is its primary orientation toward B2C scenarios, possibly overlooking the multifaceted complexities and dynamics inherent to B2B interactions.

Lin et al. (2019) introduces a groundbreaking algorithm in their study. Rooted in a Pareto efficient framework, their research emphasizes the algorithm's scalability and adaptability. However, its primary focus on multi-objective recommendation might not resonate with all application scenarios, particularly those driven by singular, focused objectives.

Li et al. (2021) embarks on a pioneering exploration into the intricate domain of false click information within E-commerce recommendation systems. Their study offers a robust set of attack detection techniques, meticulously tailored for the E-commerce landscape. However, a potential limitation of their research lies in its concentrated focus on specific attack types. This narrow lens might curtail the broader applicability of their findings in diverse E-commerce scenarios.

In practical business operations, to safeguard operational profits, it is essential to accurately identify potential churners within the customer base. Traditionally, customer churn prediction has relied on binary classifiers. However, traditional binary classifiers often overlook the costs of misclassification and the benefits of accurate classification. To enhance the accuracy of recommendation system, Jiang et al. (2024) and Liu et al. (2024) propose a profit-driven weighted classifier for customer churn prediction. The researchers develop a novel recommendation algorithm utilizing Extreme Gradient Boosting Trees to predict profit-driven customer churn, incorporating Bayesian (Liu et al., 2024) and artificial hummingbird optimization algorithm (Jiang et al., 2024). Simulations demonstrate that the new algorithm significantly improves profits compared to comparable classifiers and offers strong interpretability based on the Shapley additive explanation value (Jiang et al., 2024). Additionally, such novel recommendation system based on the Bayesian algorithm can promisingly reduce computational costs (Liu et al., 2024). Furthermore, simulations based on real data reveal that this system markedly enhances hotel profits, and sensitivity analysis indicates the new algorithm's robustness (Liu Z. et al., 2023).

In summation, the body of literature on E-commerce recommendation systems has made monumental strides, significantly advancing the field. However, a glaring gap remains in their application to specialized sectors, notably the electric power industry. This industry, characterized by its unique B2B dynamics, prolonged sales cycles, and highly specialized product offerings, presents a myriad of challenges that remain largely unaddressed in the current literature. The conspicuous absence of tailored recommendation systems for the electric power industry underscores a pressing need for dedicated research and innovative solutions for this pivotal sector.

### 2.2 Data fusion in recommendation systems

The electric power E-commerce system is inundated with a plethora of data types, from user behavior patterns, transaction histories, and product specifications to external factors like market trends and regulatory changes. The challenge lies in effectively harnessing this multifaceted data to generate actionable insights. A promising avenue that has garnered significant following in recent years is the concept of data fusion in recommendation systems, especially within the E-commerce domain.

Hu et al. (2015) showcases that CFSF outperforms existing collaborative filtering approaches in terms of recommendation accuracy and scalability. Yet, the cloud based nature of the system might raise concerns regarding data security and privacy. Jiang et al. (2020) captures the semantic access preference of users through an algorithm. The approach matches the current user session with the acquired user preference to obtain the recommendation set. The fusion of intelligent ontology and big data mining is innovative, but its complexity might pose challenges in real time recommendation scenarios.

Wróblewska et al. (2022) presents a machine learning based recommendation system that supports multiple types of interaction data with various modalities of metadata through a multimodal fusion of different data representations. While the approach is innovative, its generalizability across diverse E-commerce platforms remains to be seen. Gao and Li (2022) emphasizes the addition of semantic sentiment analysis to improve recommendation accuracy. The approach has shown a higher accuracy in recommending products users have expressed interest in. However, the model's focus on semantic sentiment might limit its broader utility.

Liu S. et al. (2023) proposes a pretrained vision and language model that integrates heterogeneous product information in a single stream Bidirectional Encoder Representations from Transformers (BERT) style architecture. This approach leverages data fusion to tap into each modality fully during pertaining. However, the model's reliance on the BERT framework might introduce complexities in the real world E-commerce applications. Ananth Gouri et al. (2023) reveals that the proposed contextual information sentiment based model outperforms the conventional collaborative filtering technique. However, the model's heavy reliance on sentiment analysis might not be suitable for all E-commerce scenarios.

In conclusion, while data fusion techniques in E-commerce recommendation systems have shown promising results, their direct applicability to specialized sectors, notably the electric power E-commerce system, remains a challenge. The unique nature of transactions, specialized product offerings, and the critical importance of accurate recommendations in the electric power industry necessitate tailored solutions that current literature has yet to address comprehensively.

### 2.3 Recommendation model integration

The integration of recommendation models in E-commerce has been a focal point for researchers and industry experts alike. As the electric power E-commerce system grapples with its unique challenges, understanding the broader landscape of recommendation model integration becomes crucial.

Wang et al. (2016) presents an innovative recommendation system that integrates pattern recognition algorithms. This model promises enhanced recommendation accuracy by analyzing user behavior preferences. Yet, the model's focus on pattern recognition might not be universally applicable across diverse E-commerce scenarios. Liu and Liu (2016) highlights the role of big data mining in understanding consumer behavior. The approach offers insights into consumer preferences, but the vastness of big data might introduce challenges in real time recommendation generation. Li et al. (2018) delves deep into potential user interests to enhance recommendation quality and real-time performance. While the approach is promising, the complexities of integrating multiple layers might pose challenges in real world applications.

Wei (2021) proposes a personalized recommendation algorithm that integrates user preference models with Back Propagation (BP) neural networks. This integrated approach promises enhanced recommendation accuracy, especially in predicting user interests. Chen et al. (2021) presents a Dual Attention Transfer based on Multi-Dimensional Integration (DAT-MDI) model. This model has shown superiority over traditional methods in various benchmark datasets. However, the integration of dual following mechanisms might introduce complexities that could hinder its applicability in certain E-commerce scenarios.

Li (2022) showcases the integration of following mechanisms with random features in a knowledge graph recommendation model. The proposed algorithm outperforms many conventional recommendation algorithms, highlighting the potential of integrated models. Bagwari et al. (2022) presents a hybrid model that integrates Decision Support Systems (DSS) with behavioral analytics. The model promises tailored recommendations, but its reliance on multiple data sources might introduce integration challenges. Cao (2022) explores the role of distributed data integration algorithms in modeling consumer behavior. The approach promises enhanced recommendation accuracy, but the complexities of distributed systems might pose challenges in real time scenarios.

In conclusion, while the integration of various recommendation models in E-commerce has shown promising results, their direct applicability to specialized sectors, notably the electric power E-commerce system, remains a challenge. The unique nature of transactions, the specialized offerings of products, and the critical importance of accurate recommendations in the electric power industry necessitate tailored solutions that the current literature has not yet addressed comprehensively.

## 3 Methodology

**Figure 2:**
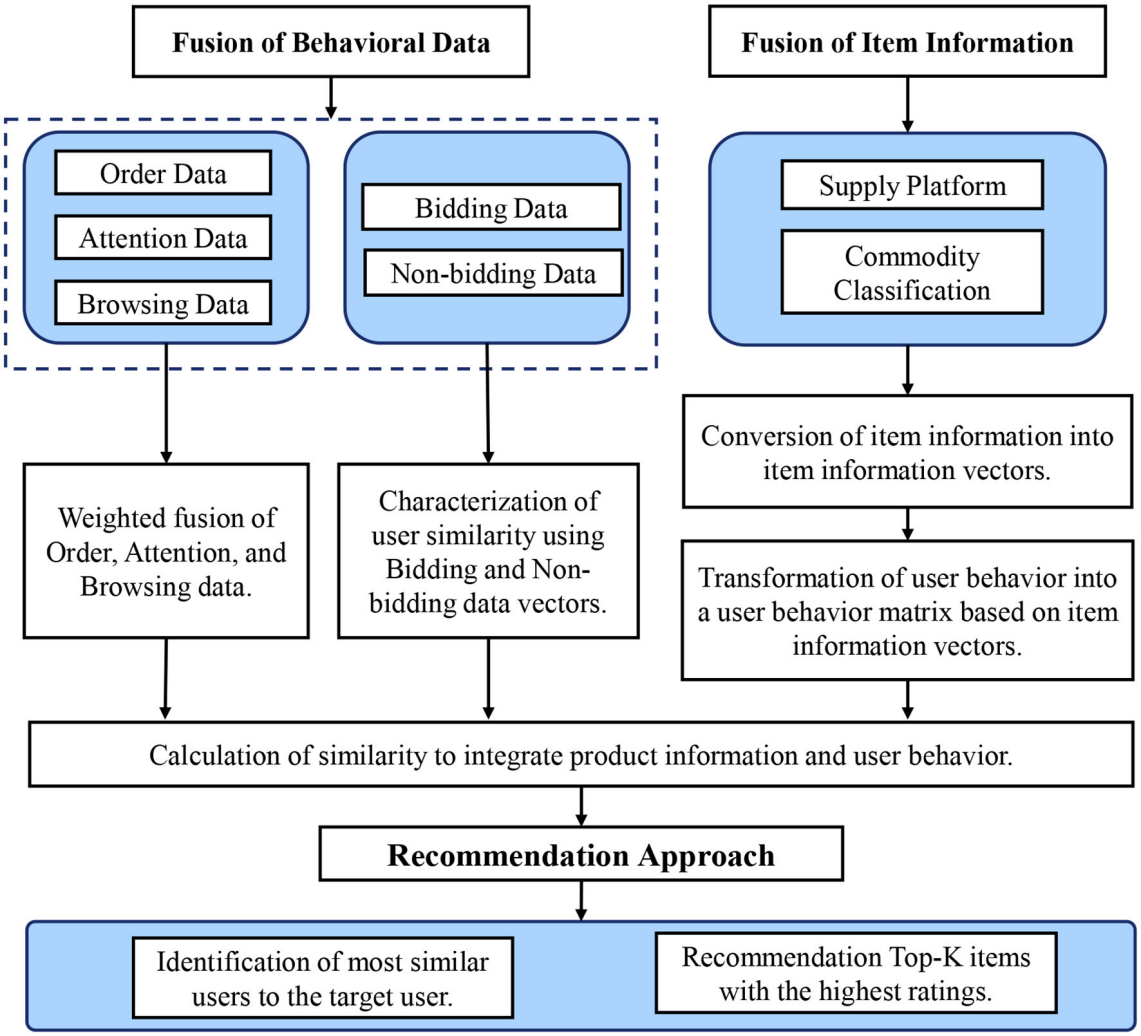
The framework for proposed recommendation system.

The methodology employed in this research is a testament to the rigorous and systematic approach adopted to address the unique challenges of the electric power E-commerce domain. The methodological framework is rooted in the synthesis of behavioral data and item attribute information, culminating in a recommendation method that is both robust and tailored to the specific nuances of the industry. This section elucidates the intricacies of this approach, detailing each component and the underlying rationale. The comprehensive framework of the methodology is depicted in Figure 2.

### 3.1 Electric power B2B descriptions

The Business-to-Business (B2B) framework within the electric power industry stands as a unique and complex entity, markedly different from the more familiar terrain of consumer focused markets. This distinction is not merely in scale but also in the depth and intricacies of its operations. Predominantly characterized by transactions of substantial magnitude, the electric power B2B sector encompasses a vast array of activities. These range from the procurement of heavy duty equipment in bulk quantities to the formulation and execution of comprehensive contracts that oversee power generation, transmission, and distribution across vast geographical expanses.

The sheer scale and complexity of these transactions give rise to extended sales cycles. Unlike the swift decision-making often seen in B2C scenarios, B2B dealings in the electric power industry are methodical and deliberate. Each transaction, be it a purchase order for machinery or a long-term service contract, undergoes a meticulous process of evaluations. These assessments are not just about cost-effectiveness but also delve into the technical compatibility, long-term viability, and potential scalability of the offerings. Negotiations, often spanning multiple rounds, aim to strike a balance between commercial interests and operational feasibility. Furthermore, the approval processes are multi-tiered, involving various stakeholders from technical experts and financial analysts to top-tier management, ensuring that every decision aligns with the organization's strategic objectives.

In this industry, the products and services on offer are not generic; they are highly specialized solutions crafted to address specific challenges. Whether it's a state-of-the-art transformer, an advanced grid management system, or consultancy services for renewable energy integration, each product or service demands a deep-seated understanding. Stakeholders must grasp not only the technical specifications but also the broader implications of their choices. This includes potential integration challenges with existing systems, adherence to ever-evolving regulatory frameworks, and ensuring compliance with both local and international safety and environmental standards. In essence, every B2B transaction in the electric power sector is a testament to the industry's multifaceted nature, where commercial, technical, and regulatory considerations converge.

### 3.2 Notations

In this section, we introduce and define the notations that will be used throughout the methodology. This notation serves as the foundation for understanding the data structures, user behaviors, and item attributes, as well as the collaborative filtering approach we use to recommend products to users.

*U:* Represents the set of all users in the system. Each user is uniquely identified by an index in this set, *N* represents the number of the users.

*I:* Denotes the set of all items available in the system. Similar to users, each item is uniquely identified by an index in this set, *M* represents the total number of the items.

*n*: Represents the number of categories for individual product attributes.

*m*: Represents the number of categories for user behaviors.

*S* (*u, v*)*:* Represents the similarity between users *u* and *v*. This similarity metric is crucial for collaborative filtering, as it determines how similar two users are in terms of their preferences. The basic formula for collaborative filtering is given by Equation 1:


(1) $$\begin{array}{l} r_{ui}=\underset{v\in U}{\sum }S\left(u,v\right)r_{vi} \end{array}$$

*O*_*ui*_, *A*_*ui*_, *B*_*ui*_: these notations denote the order, following, and browsing numbers respectively for user *u* and item *i*. They capture different types of implicit feedback from users.

*w*_*o*_, *w*_*a*_, *w*_*b*_: these are the weight ratios associated with order, following, and browsing data respectively. They determine the significance or influence of each type of implicit feedback in the recommendation process. For examples, we can set *w*_*o*_ = 1, *w*_*a*_ = 0.5, *w*_*b*_ = 0.5.

*BI*_*u*_, *NBI*_*u*_: these vectors are behavior numbers varying time, representing the bidding and non-bidding behaviors of user *u* respectively. They capture unique B2B behaviors that are essential for understanding user preferences in the electric power E-commerce domain.

The foundation of this collaborative filtering approach is based on the principle that users who have behaved similarly in the past will continue to have similar preferences in the future. The notations introduced above will be instrumental in formulating and understanding the mathematical models and algorithms we employ in subsequent sections.

### 3.3 Fusion of behavioral data

The fusion of behavioral data is a pivotal step in the methodology, aiming to create a comprehensive representation of user interactions on the platform. This section delves into the intricacies of how different types of behavioral data are combined to provide a holistic view of user preferences and activities.

#### 3.3.1 Order, following, and browsing data

These three types of data capture the most direct interactions of users with items on the platform. Order data (*O*_*ui*_) represents confirmed transactions, following data (*A*_*ui*_) signifies items that users have shown interest in, and browsing data (*B*_*ui*_) captures the items that users have viewed or explored.

#### 3.3.2 Weight ratios

The weight ratios *w*_*o*_, *w*_*a*_*, and* *w*_*b*_ are crucial in determining the significance of each type of interaction. They allow us to assign varying importance to different behaviors, reflecting the relative impact of each interaction type on user preferences. For instance, a confirmed order might carry more weight than merely browsing an item.

#### 3.3.3 Bidding and non-bidding data

Unique to the B2B E-commerce landscape, bidding (*BI*_*u*_) and non-bidding (*NBI*_*u*_) data provide insights into the negotiation and decision-making processes of users. These behaviors, while not directly linked to transactions, offer valuable context about user intentions and preferences.

#### 3.3.4 Behavior vectors

The behavior vectors for bidding and non-bidding data are formulated to capture the essence of these unique interactions. By characterizing user similarity through these vectors, we can better understand the relationships and similarities between users based on their bidding and non-bidding behaviors.

The fusion formula for order, following, and browsing data is given by Equation 2:


(2) $$\begin{array}{l} F_{ui}\;=\;w_{o}O_{ui}\;+\;w_{a}A_{ui}\;+\;w_{b}B_{ui} \end{array}$$

This formula ensures that each type of interaction contributes proportionally to the final fused representation based on its assigned weight. A cosine similarity measure is used here to characterize user similarity for the fusion of behaviors, as detailed in Equation 3.


(3) $$\begin{array}{l} S_{f}\left(u,v\right)=\cos\left(\theta \right)=\frac{F_{u}\cdot F_{v}}{\left||F_{u}||||F_{v}|\right|} \end{array}$$

For bidding and non-bidding data, we employ a cosine similarity measure to characterize user similarity *S*_*bid*_(*u, v*), *S*_*nb*_(*u, v*). This metric captures the angle between the behavior vectors, providing a measure of how alike two users are in terms of their bidding and non-bidding behaviors:

The fusion of behavioral data is a multi-faceted process that combines various types of user interactions to create a unified representation. This representation serves as the foundation for subsequent recommendation processes, ensuring that all relevant user behaviors are considered.

### 3.4 Fusion of item attribute information

The integration of item attribute information is a cornerstone in the methodology, ensuring that the attributes and characteristics of items are adequately represented and factored into the recommendation process. This section elucidates the methods and rationale behind the fusion of various item related data, highlighting the importance of capturing the multifaceted nature of products in the B2B E-commerce domain.

#### 3.4.1 Item attribute vector

Central to the approach is the concept of item attributes. This vector encapsulates various attributes of an item, such as its category, suppliers and other relevant metadata. For each item and each item attribute (e.g. category), we construct a one-hot-vector based on different values of attributes. Hence, the size of item attribute vector *IA* related to individual item attribute is *M* × *n*.

#### 3.4.2 User behavior matrix

With the item attribute vectors in place, we can then construct the user-attribute behavior matrix. Firstly, the overall user behavior vector *B*_*m*×*M*_ is established with each element representing the number of specific behavior (e.g., order number) for each item. The *m* is the number of behavior types and *M* is the number of items. Secondly, through matrix operations *B*_*m*×*M*_*IA*_*M*×*n*_, we can obtain the user specific-attribute interaction matrix *R*_*m*×*n*_. Finally, by summing up each column of the matrix, we can obtain a vector representing the behavioral performance of each user for each attribute. Therefore, the user-attribute behavior matrix *M*_*N*×*n*_ is established. Mapping user behaviors onto the item attribute vectors, we can capture the nuanced interactions between users and the multifaceted attributes of items.

#### 3.4.3 Similarity computation

The fusion of item attribute information and user behavior culminates in the computation of similarity scores. The item-attribute similarity *S*_*item*_(*u, v*) extracted from *M*_*N*×*n*_, measure the likeness between two users based on their item attribute information (e.g. item category, suppliers) and the aggregated user interactions (e.g., order, following, browsing). The similarity scores play a pivotal role in the recommendation process, guiding the system toward items that are contextually and attribute wise similar to the user's preferences.

To compute the similarity between users based on their item attribute vectors and user interactions, we employ the cosine similarity metric. In this paper, *S*_*item*_(*u, v*) is composed of four parts, mainly the three category (primary, secondary and tertiary item category) similarities *S*_*ite*_*m*__*category*__(*u, v*) and one supplier similarity *S*_*ite*_*m*__*supplier*__(*u, v*).

In essence, the fusion of item information is a meticulous process that aims to bridge the gap between raw item attributes and structured data representations. By integrating item characteristics with user behaviors, we ensure that the recommendation system is both context-aware and attribute-sensitive, leading to more accurate and meaningful recommendations.

### 3.5 Fusion of behavioral data and item information

The recommendation method forms the crux of the approach, leveraging the fused behavioral data and item information to generate personalized product suggestions for users. This section delves into the intricacies of the recommendation algorithm, elucidating the steps and logic that drive the generation of tailored recommendations.

#### 3.5.1 User similarity computation

At the heart of the recommendation method is the computation of user similarity. By comparing the behavior profiles of different users, we can identify patterns and preferences that are shared among them. This similarity metric, denoted as *S*(*u, v*), provides a measure of how alike two users are in terms of their interactions and preferences. It serves as a foundation for identifying potential items that might be of interest to a given user, as detailed in Equation 4.


(4) $$\begin{array}{l} S\left(u,v\right)=S_{f}\left(u,v\right)+S_{bid}\left(u,v\right)+S_{nb}\left(u,v\right)+S_{item}\left(u,v\right) \end{array}$$

The elements in similarity matrix *S*(*u, v*) vary between 0 and 7. The higher the value in the matrix, the greater the similarity between the related users.

#### 3.5.2 Historical data consideration

A user's historical data plays a pivotal role in shaping recommendations. By analyzing past interactions, purchases, and preferences, we can glean insights into a user's tastes and inclinations. This historical context ensures that the recommendations are not only based on current interactions but also influenced by a user's long-term behavior.

#### 3.5.3 Item ranking

Once user similarities are computed and historical data is factored in, the next step is to rank items based on their relevance to a given user. This ranking process involves scoring items based on their potential appeal to the user, considering both the user's behavior and the item's attributes.

#### 3.5.4 Top-K recommendations

The culmination of this recommendation method is the generation of the Top-K recommendations. These are the K items that have the highest relevance scores for a user. By focusing on the top-rated items, we ensure that users are presented with products that are most likely to align with their preferences and needs.

The recommendation for a user *u* is formulated as Equation 5:


(5) $$\begin{array}{l} RS_{u}=\left\{i_{1},\;i_{2},\;\ldots ,\;i_{K}\right\} \end{array}$$

where *i*_*k*_ ∈ *I* , and *K* is defined as the mean of historical order number of user *u*. This formula ensures that the recommended items are those with the highest relevance scores, taking into account both user similarity and item attributes.

In summary, this recommendation method is a multi-faceted approach that synergizes user behaviors, item attributes, and historical data to generate personalized product suggestions. By considering a wide array of factors and employing sophisticated algorithms, we aim to provide users with recommendations that are both relevant and meaningful, enhancing their e-commerce experience.

## 4 Experiments and discussion

In this section, a real case dataset was utilized to assess the effectiveness of the proposed recommendation model. The case study results demonstrate the proficient performance of the proposed approach.

### 4.1 Data descriptions

The research presented in this study heavily relies on the rich dataset sourced from Beijing Huadian E-commerce Technology Limited Company, a leading entity in the B2B E-commerce sector of the electric power industry. This meticulously curated dataset, which spans the entire duration of 2022, offers an unparalleled window into the multifaceted interactions of 217 distinct users as they navigate through an extensive catalog of 346,672 products.

At the heart of this dataset lies five pivotal categories of user behavior: order data, following data, browsing data, bidding data, and non-bidding data. Each of these categories, while valuable in its own right, collectively paints a comprehensive picture of user interactions, preferences, and decision-making processes on the platform. The more traditional data types, such as order, following, and browsing data, provide insights into patterns of product discovery, interest, and acquisition. On the other hand, the inclusion of specialized data types like bidding and non-bidding data offers a deep dive into the unique B2B behaviors that set this platform apart from conventional e-commerce platforms. The intricate relationship between bidding activities and order behaviors, for instance, sheds light on the multi-layered negotiation, evaluation, and decision-making phases that often precede a finalized B2B transaction.

#### 4.1.1 Product categories

There are 18 primary categories, as well as over 1,900 sub-categories of products in this dataset, each reflecting a specific segment of the electric power industry's vast product landscape. These categories not only signify the depth and breadth of products available on the platform but also highlight the diverse needs and preferences of the B2B clientele. A detailed enumeration of the most dominant categories, based on the sheer volume of products, is systematically presented in Table 1. In this article, the products themselves, rather than the product categories, are the target of our recommendations. We have incorporated the product classification information (primary, secondary and tertiary item category) into the recommendation model when constructing item-attribute similarities.

**Table 1:** Breakdown of prominent product categories.

**Product category**	**Number of products**
Office supplies	243,430
Tools	47,337
Labor protection	30,540
Sealing	18,642
Electrician tools	17,159
Fastening	16,793
Pipes and valves	15,103
Storage and transport	15,076
Instruments	14,609

#### 4.1.2 Suppliers

The dataset's richness is further amplified by contributions from nine distinguished suppliers, each renowned for their significant product offerings and industry reputation. These suppliers, with their varied product portfolios, play a crucial role in shaping the B2B e-commerce ecosystem of the electric power industry. A comprehensive overview of these major suppliers, along with a breakdown of their product contributions, is encapsulated in Table 2. In this paper, supplier information is also employed to compute item-attribute similarities.

**Table 2:** Major suppliers and their product contributions.

**Supplier name**	**Number of products**
HuaDian Group	160,879
JD century information Tech	123,154
Western wisdom supply chain	52,749
Morning light office supplies	46,237
Zhenkun industrial supermarket	36,839

#### 4.1.3 User behavior data

To delve deeper into the user centric aspect of the dataset, a granular breakdown of the user behavior categories, complemented by the number of records associated with each, is meticulously detailed in Table 3. The industry classification of the 217 unique users is primarily concentrated in the electricity, heat, gas and water production major categories. This segmentation offers an in depth perspective into user interactions, shedding light on their preferences, behaviors, and purchasing patterns, thereby enabling a nuanced exploration of the underlying dynamics of B2B e-commerce within the electric power industry. The user behavioral data comprises a variety of metrics, including order records, following actions, browsing histories, bidding and non-bidding records. Among these, the follow behavior data is relatively sparse, while the order records and browsing histories constitute a comparatively larger volume of data. The total order volume for the year 2022 was 324,001, with a total of 217 users. Typically, an order contains only one type of product. Thus, the average annual order volume per user is around 1,493, with an average monthly order frequency of ~124 times. This is highly relevant to the type of institutional users. For consumable products, users may concentrate their purchases from different suppliers over a certain period, resulting in a higher order volume.

**Table 3:** Breakdown of user behavior data categories.

**User behavior type**	**Number of records**
Order data	324,001
Following data	307
Browsing data	1,048,575
Bidding data	11,595
Non-bidding data	99,025

### 4.2 Comparison criteria

In the case study, the primary objective is to evaluate the effectiveness of this recommendation system by assessing the accuracy of the recommended items in relation to the items that users have actually purchased. To achieve this, we employ a set of well-established metrics: Precision (*P*_*u*_), Recall (*R*_*u*_), and the *F*_1_ score (*F*_1, *u*_), based on the Recommendation Set (*RS*_*u*_) and Purchased Set (*PS*_*u*_).

#### 4.2.1 Recommendation set (**RS**_**u**_)

This set comprises the items that this recommendation system suggests for a user *u*. For instance, if the system recommends 100 items, *RS*_*u*_ will contain these 100 elements.

#### 4.2.2 Purchased set (**PS**_**u**_)

*PS*_*u*_ represents the set of items that user *u* has actually purchased. This set serves as the ground truth against which the recommendations are compared.

#### 4.2.3 Precision (**P**_**u**_)

Precision evaluates the accuracy of the recommendations by measuring the proportion of recommended items that the user has actually purchased. Mathematically, it is defined as in Equation 6:


(6) $$\begin{array}{l} P_{u}=\frac{\left|RS_{u}\cap PS_{u}\right|}{\left|RS_{u}\right|} \end{array}$$

A higher *P*_*u*_ indicates that a larger fraction of the recommended items aligns with the user's actual purchase behavior, signifying more accurate recommendations.

#### 4.2.4 Recall (**R**_**u**_)

Recall assesses the completeness of the recommendations by determining the proportion of items that the user purchased which were also recommended by the system. It is given by Equation 7:


(7) $$\begin{array}{l} R_{u}=\frac{\left|RS_{u}\cap PS_{u}\right|}{\left|PS_{u}\right|} \end{array}$$

A higher *R*_*u*_ suggests that the recommendation system effectively captures the user's purchasing tendencies.

#### 4.2.5 **F**_**1**_ score (**F**_**1, u**_)

The *F*_1_ score is the harmonic mean of Precision and Recall, providing a single metric that balances the trade-off between the two. It is defined as in Equation 8:


(8) $$\begin{array}{l} F_{1,u}=\frac{2P_{u}R_{u}}{P_{u}+R_{u}} \end{array}$$

A higher *F*_1, *u*_ score indicates a better overall performance of the recommendation system.

In essence, these metrics allow us to quantitatively evaluate the performance of this recommendation system, ensuring that the suggested items are both accurate (reflecting the user's actual purchases) and comprehensive (capturing the breadth of the user's purchasing behavior).

### 4.3 Comparison results

In this comprehensive comparative analysis, we pose the proposed recommendation method against two other widely recognized methods: BPR (Rendle et al., 2012) and BiVAE (Truong et al., 2021). The overarching aim is to delineate the distinctive advantages and superior performance of this approach, shedding light on its potential to revolutionize the electric power E-commerce domain. Moreover, we implement ablation experiments for different behavior data and different similarity terms, respectively.

**Figure 3:**
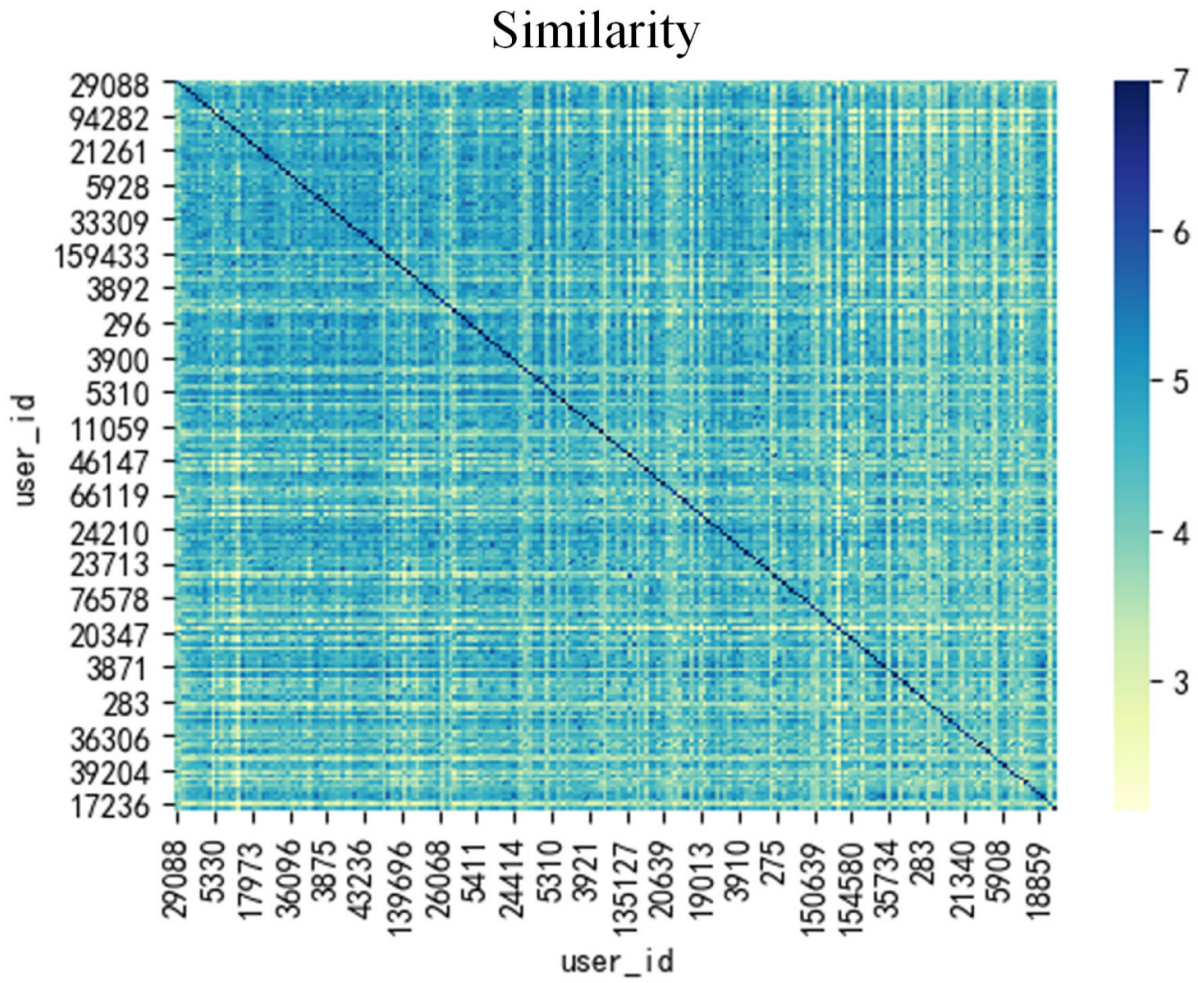
The heat map for user similarity based adopted data.

As shown in Table 4, the precision of our proposed method has reached 0.028, which is three times higher than the traditional method's 0.0075 and twice as high as 0.014, demonstrating the superiority of our approach. Tables 5, 6 and Figure 3 illustrate the similarity of users in the proposed method. The model result offers a panoramic view of the performance metrics, painting a vivid picture of the strengths and capabilities of proposed method. A cursory glance reveals the undeniable edge the method holds, especially in terms of precision. A heightened precision exhibited by this method is a testament to its adeptness at discerning and catering to user preferences. The recall metric further amplifies the method's prowess. This method's superior recall rate underscores its holistic approach, ensuring that a vast majority of user purchases find representation in the recommendations. The unrivaled *F*_1, *u*_ score accentuates its balanced and nuanced approach, setting it leagues apart from its counterparts.

**Table 4:** Comparison of the proposed method against other prevalent recommendation techniques.

**Method**	**Precision**	**Recall**	**F_1_ score**
Proposed method	0.028400	0.045111	0.031458
BPR (Li, 2022)	0.007514	0.013120	0.008717
BiVAE (Bagwari et al., 2022)	0.014587	0.022839	0.015696

**Table 5:** Ablation experiment results for different behavioral data.

**Method**	**Precision**	**Recall**	**F_1_ score**
Order + following + browsing	0.028400	0.045111	0.031458
Order + browsing	0.028605	0.045179	0.031636
Order + following	0.028049	0.044651	0.031272
Order	0.028399	0.045476	0.031658

**Table 6:** Ablation experiment results for different similarity terms.

**Method**	**Precision**	**Recall**	**F_1_ score**
All	0.028400	0.045111	0.031458
Basic cosine+ bidding + non-bidding	0.028138	0.044011	0.030947
Basic cosine + bidding	0.027651	0.043794	0.030536
Basic cosine	0.026532	0.041283	0.029012

Diving deeper into the intricacies of the electric power E-commerce domain, one encounters a labyrinth of challenges. The unique nature of transactions, the specialized product range, and the intricate user behaviors make the task of recommendation a Herculean challenge. Yet, this method, armed with its innovative algorithms and data fusion techniques, navigates this maze with unparalleled finesse. Its stellar performance, as evidenced by the comparison, is not just a testament to its technical brilliance but also its adaptability to the domain's idiosyncrasies.

In wrapping up this analysis, the results resoundingly champion the cause of this recommendation system. Its consistent and dominant performance over established methods not only carves a niche for it in the annals of e-commerce recommendations but also promises transformative benefits for users and businesses alike in the electric power industry. As the industry continues to evolve, this method stands poised to lead the charge, setting new benchmarks and redefining excellence.

As industries continue to digitize, this method offers promising benefits for users and businesses alike. In future work, the following areas can be improved:

I) Enhanced Data Preprocessing and Feature Engineering: We acknowledge the importance of sophisticated data preprocessing and feature engineering. Moving forward, we plan to incorporate more features of items with the existing description text and images, as well as automated feature engineering techniques to uncover latent data patterns, thereby boosting the model's predictive accuracy. Furthermore, we intend to implement advanced data augmentation methods, including simulation of user interactions in sparse data scenarios, to deepen the model's comprehension of user behaviors and preferences.
II) Tackling Long-Tail and Cold-Start Issues: We recognize the critical nature of long-tail distribution and cold-start challenges in recommendation systems. To address these, our future work will explore the integration of auxiliary user and product information, leveraging insights from social media analyses and user feedback. We aim to significantly enhance the system's recommendation quality for new users and products, effectively mitigating these challenges.
III) Cross-Domain Model Generalization: The feedback on testing our model's generalization across different industries and datasets is invaluable. We plan to conduct extensive cross-domain experiments to evaluate the model's adaptability and flexibility. This will likely involve the development of domain adaptation algorithms to tailor the model's parameters to specific industry challenges and requirements, ensuring broad applicability and robust performance across various e-commerce platforms.
IV) Real-Time Recommendation System Optimization: The suggestion to optimize the real time performance of our recommendation system resonates with our objectives. We are committed to exploring the latest in machine learning and big data technologies to improve computational efficiency for near-real-time recommendations. This will include leveraging distributed computing to manage large-scale data and developing lightweight model architectures for rapid adaptation to user behavior changes, maintaining high-quality recommendations.

## 5 Conclusion

The evolution of E-commerce in specialized sectors like the electric power industry demands advanced recommendation systems. This research presented a tailored recommendation system for this niche domain, demonstrating significant advantages over existing methods. The method consistently outperformed three other techniques across key metrics. Its precision and recall rates underscore its ability to understand user behavior and offer comprehensive recommendations. The harmonized *F*_1, *u*_ score further attests to its balanced approach. The challenges of the electric power e-commerce landscape are manifold, yet this method, with its innovative algorithms and data fusion techniques, has proven adept at navigating them. The case study validated the practical applicability of this approach.

Through the strategic utilization of multidimensional data and sophisticated machine learning methodologies, our approach have elevated the precision of product recommendations. Consequently, this enhancement could optimize the efficiency and effectiveness of B2B transactions. In its entirety, this newly developed approach endeavors to establish a novel benchmark in E-commerce recommendations tailored to industries characterized by distinctive transactional attributes, which help provide the groundwork for the formulation of more specialized and effective recommendation strategies.

Potential directions for future research could delve into the scalability of this method across other specialized industries, explore enhancements in data fusion techniques, and investigate potential integrations with emerging technologies to further optimize recommendation accuracy and user experience.

## Data availability statement

The data analyzed in this study is subject to the following licenses/restrictions. The data is available after acceptable upon reasonable request. Requests to access these datasets should be directed at: chenlili1002@126.com.

## Author contributions

WM: Writing – review & editing, Writing – original draft, Visualization, Methodology, Formal analysis, Data curation. LC: Supervision, Methodology, Investigation, Formal analysis, Conceptualization, Writing – review & editing. ZD: Validation, Data curation, Writing – review & editing.
